# Complications in the use of the mandibular body, ramus and symphysis as donor sites in bone graft surgery. A systematic review

**DOI:** 10.4317/medoral.20938

**Published:** 2016-01-31

**Authors:** David Reininger, Carlos Cobo-Vázquez, Marta Monteserín-Matesanz, Juan López-Quiles

**Affiliations:** 1 DDS, Master in Oral Surgery and Implantology, Complutense University of Madrid; 2 MD, DDS, PhD, Maxillofacial Surgeon, Associate Professor, Complutense University of Madrid. Department of Oral Surgery and Maxillofacial Surgery, Complutense University of Madrid, Spain

## Abstract

**Background:**

To develop a systematic review by assessing and comparing the different complications that occurs in bone graft surgery using the mandibular body, ramus and symphysis as donor sites.

**Material and Methods:**

In order to respond to the following question, a systematic review was developed: does the use of intraoral mandibular body and ramus as donor sites in bone graft surgery, produce fewer and less severe complications in comparison to the use of the mandibular symphysis in patients that present bone resorption that needs augmentation using autologous grafts? The review was carried out between January 1990 and 2015, during which only clinical essays with a minimum follow-up period of six months were included.

**Results:**

The initial search yielded a total of 2912 articles, of which 6 were finally selected. In total, 259 graft surgeries were performed; 118 using the mandibular body and ramus as donor sites, and 141, the symphysis. The most frequent complications that arose when using the mandibular symphysis were temporary sensory alterations in the anterior teeth (33.87%), followed by sensory alterations of the skin and mucosa (18.57%). As for the mandibular body and ramus donor sites, the most frequent complications relate to temporary sensory alterations of the mucosa (8.19%) and to minor postoperative bleeding (6.55%).

**Conclusions:**

The analyzed results show a higher prevalence and severity of complications when using mandibular symphysis bone grafts, producing more discomfort for the patient. Therefore, it would be advisable to perform further clinical essays due to the lack of studies found.

**Key words:**Alveolar ridge augmentation, autogenous bone, mandibular bone grafts, chin, mandibular symphysis, mandibular ramus.

## Introduction

The implant management of the partial or completely edentulous patient is nowadays a daily and routine treatment with proven success rates in the long term (1). To achieve functional and aesthetic success of the implant, a specific minimum of requirements are taken into consideration. Bone quantity (in terms of both thickness and length), and an acceptable bone quality, are necessary (2) to achieve the stability of the implant. However, there are local conditions such as agenesis, early dental extraction, trauma, lack of facial development or surgical resection that can lead to a decrease in bone volume, which would make the correct placement of the implant impossible, having to resort to bone regeneration techniques.

Among the described bone volume restoration procedures (2), the use of the following are included: onlay grafts, inlay grafts, bone expansion, guided bone regeneration and alveolar distraction osteogenesis. The majority of techniques (excluding alveolar distraction osteogenesis) require the use of autologous grafts or other types of bone substitute, xenografts, allografts or alloplastic grafts (1,2). In spite of the wide variety of existing alternatives, the autologous graft is still generally considered as the most effective, with success rates as high as 95%, considered as “the gold standard” (3,4) among the different types of grafts due to their lack of immunological reactions, their osteoinductive and osteoconductive properties, and the presence of osteoprogenitor cells along with growth factors (3).

Autologous grafts can be classified as extraoral or intraoral (3). Intraoral grafts offer the following advantages (3,5,6): proximity to the receptor site, reduction of operating time and a considerable amount of local anaesthesia, eliminating the need of hospitalization, and finally, lower morbidity and discomfort for the patient. On the other hand, the main disadvantages (3,6,7) using these grafts are the postoperative effects that may occur, among which the following are described: dental, mucosal or skin sensory disturbances, limitation of the opening and alteration of the facial contour. Regarding the use of these grafts, Schwartz-Arad and Levin (8) concluded that intraoral bone graft surgery is considered as a predictable surgical procedure with a high success rate.

There are multiple donor sites described in the literature that can be used as bone grafts. Among them are: the mandibular body and ramus (9), the symphysis (10), the coronoid processes (11), the anterior maxillary sinus wall (12), the tuberosity (13), the zygoma bone (14), tori (15), and the anterior nasal spine (16). Of all the mentioned areas, the most commonly used donor sites are the mandibular body, ramus and symphysis. Comparing these grafts, it could be said that on one hand, the body and ramus bone (17,18) is dense, with less amount of marrow, providing enough bone quantity for defects of 1-3 teeth requiring a period of 4-6 months for integration, presenting minimal resorption. On the other hand, the symphysis area is described (10,19-21) as an easily accessible area, with a suitable cortical and cancellous bone volume and higher cancellous bone mass, and may grant an amount of bone for defects of 1-6 teeth, but presents an increased bone resorption.

Although there are several articles regarding the complications that follow graft surgery, few compare in the same study the complications that arise from the use of one type of graft to the other. This fact will not help the professional in making the decision of what type of graft is the most suitable, bearing in mind that it is a common surgical procedure. Therefore, the purpose of this study is to conduct a systematic review by evaluating and comparing the various complications that occur in the mandibular body, ramus and symphysis grafts as donor sites.

## Material and Methods

A protocol was developed prior to the review covering all aspects regarding the systematic review methodology as per Prisma guidelines (22), including the following definitions:

Question at issue: ¿Does the use of mandibular ramus intraoral bone grafts produce fewer and less severe complications in patients that present bone resorption that needs augmentation using autologous grafts, compared to mandibular symphysis bone grafts?

Population to be studied: The population of interest in this review corresponds to patients presenting bone resorption in need of restoration of bone volume using intraoral autologous bone grafts.

Type of intervention and comparison: The type of intervention to be studied in the review was bone graft surgery using intraoral autologous grafts extracted from the mandibular body or ramus, being compared to bone graft surgery where the graft is also of intraoral autologous origin, but obtained from the symphysis.

Selection of studies: The selected studies included clinical trials on humans during a minimum follow-up period of six months. The area from where the graft was obtained was clearly identified as well as the complications that occurred in the donor site following the grafting, specifying how the complication was measured (whether it was by a questionnaire, clinical examination, a visual analogue scale, the Two-Pointed Blunt Test, pulp vitality tests or X-rays).

All clinical trials that included patients with the following conditions were automatically excluded from the review: uncontrolled metabolic illnesses, head radiotherapy in the past 24 months, intraoral or intravenous bisphosphonate treatment during a period that exceeded 3 years, patients with mental disorders, heavy drinkers and/or smokers (more than 10 cigarettes per day), or drug addicts.

Results: The resulting complications in bone graft surgery using the mandibular body and ramus and symphysis as donor sites in the form of block or particulate grafts were the variables to be analyzed, paying more attention to the following complications: pain, postoperative hemorrhage, sensory alterations (hypoesthesia, hyperesthesia, paraesthesia, dysesthesia) in skin, mucosa or teeth, infection, pulp necrosis and mandibular fractures.

Search Strategy: The search and following selection of articles was performed by using 4 electronic databases (Pubmed, Cochrane Central Register of Controlled Trials, Ebsco and Google Scholar). Only the articles which complied with the inclusion criteria and written between January 1990 and 2015 were considered.

The search was completed using the following terms: “Population AND Intervention AND Outcomes” ( Table 1).

**Table 1:**
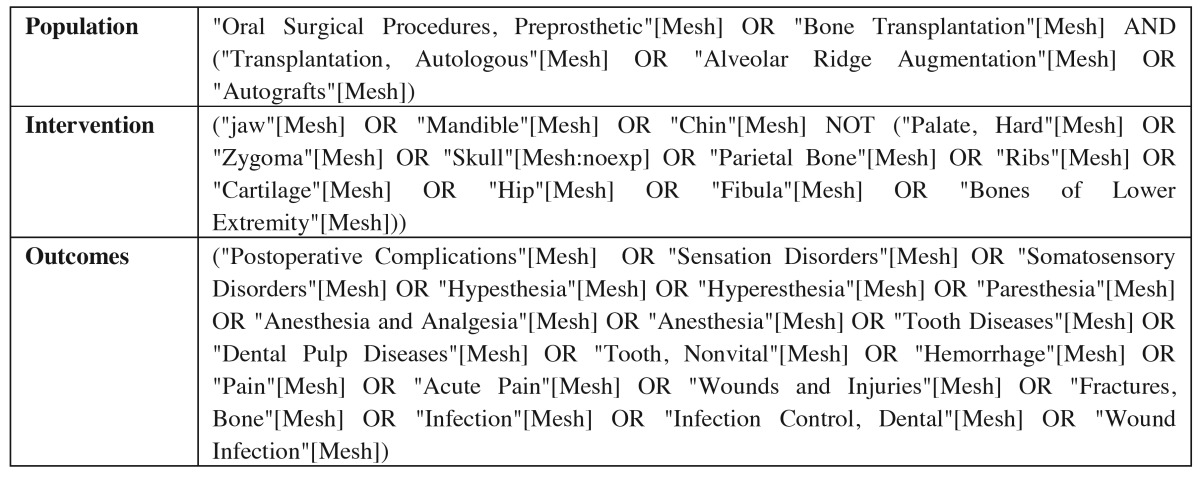
Search Strategy.

Only the articles written in Spanish or English were included. Revision of the cross-references of the initial list of selected references was carried out.

Manual search in the following journals was developed: Journal of Oral and Maxillofacial Surgery, International Journal of Oral and Maxillofacial Surgery, Clinical Oral Implants Research, Clinical Implant Dentistry and Related Research, Journal of Oral Implantology and International journal of Oral and Maxillofacial Implants.

Review Methodology: Following the initial search, the review of the resulting titles and abstracts was performed by two independent reviewers (D.R. and C.C.). Only the studies which complied with the requested inclusion criteria were included; articles whose title and/or abstract did not clearly match with the inclusion criteria were subsequently read in full text. Thus, a complete list of full text articles was obtained for the reviewers to read and select. In cases of disagreement between reviewers, a third reviewer was consulted (J.LQ.). To avoid selection bias, the reviewers concealed the name of the Journal, the institution of origin and the names of the authors of the articles during the review. Special attention was paid to duplicate publications.

Quality assessment

Following the Cochrane recommendations “assessing risk of bias in included studies” (23), the quality assessment was carried out by two independent reviewers (D.R. and C.C.).

## Results

**Figure 1:**
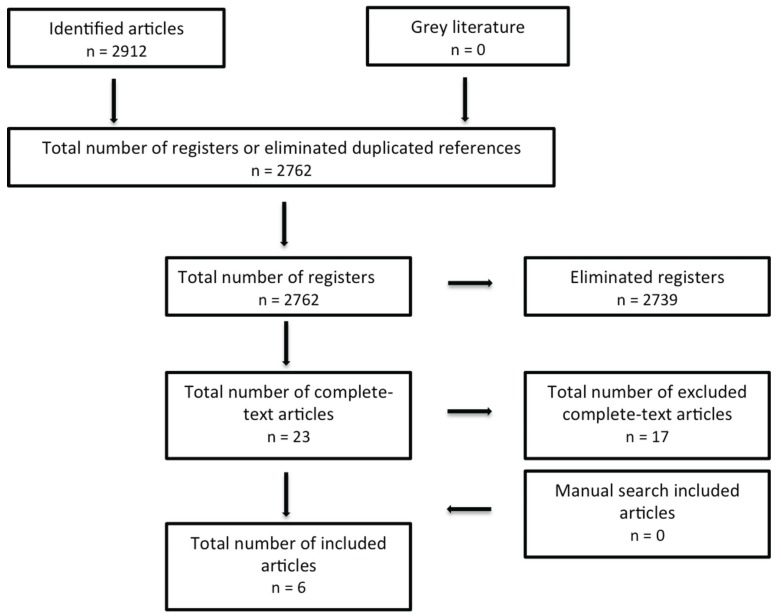
Flow diagram (based on PRISMA format) on the research and selection process.

The initial search yielded a total of 2912 articles (Fig. 1), where 23 relevant full text publications were identified. In this initial screening a match percentage of 96.25% among reviewers was obtained. Of the 23 selected publications, 16 did not meet the required inclusion criteria and were therefore excluded, leaving only 6 articles included in the review (match percentage of 97.7% among researchers). The search was supplemented with a manual review, in which no publications were contemplated (Fig. 1).

Of the 6 selected articles, the main author coincided in 2 ( Table 2 and  Table 2continue), corresponding to different investigation groups. Therefore, the articles belonged to six independent investigation groups, two from Italy, one from Kuwait, one from Holland, one from Spain and two from the United States.

**Table 2:**
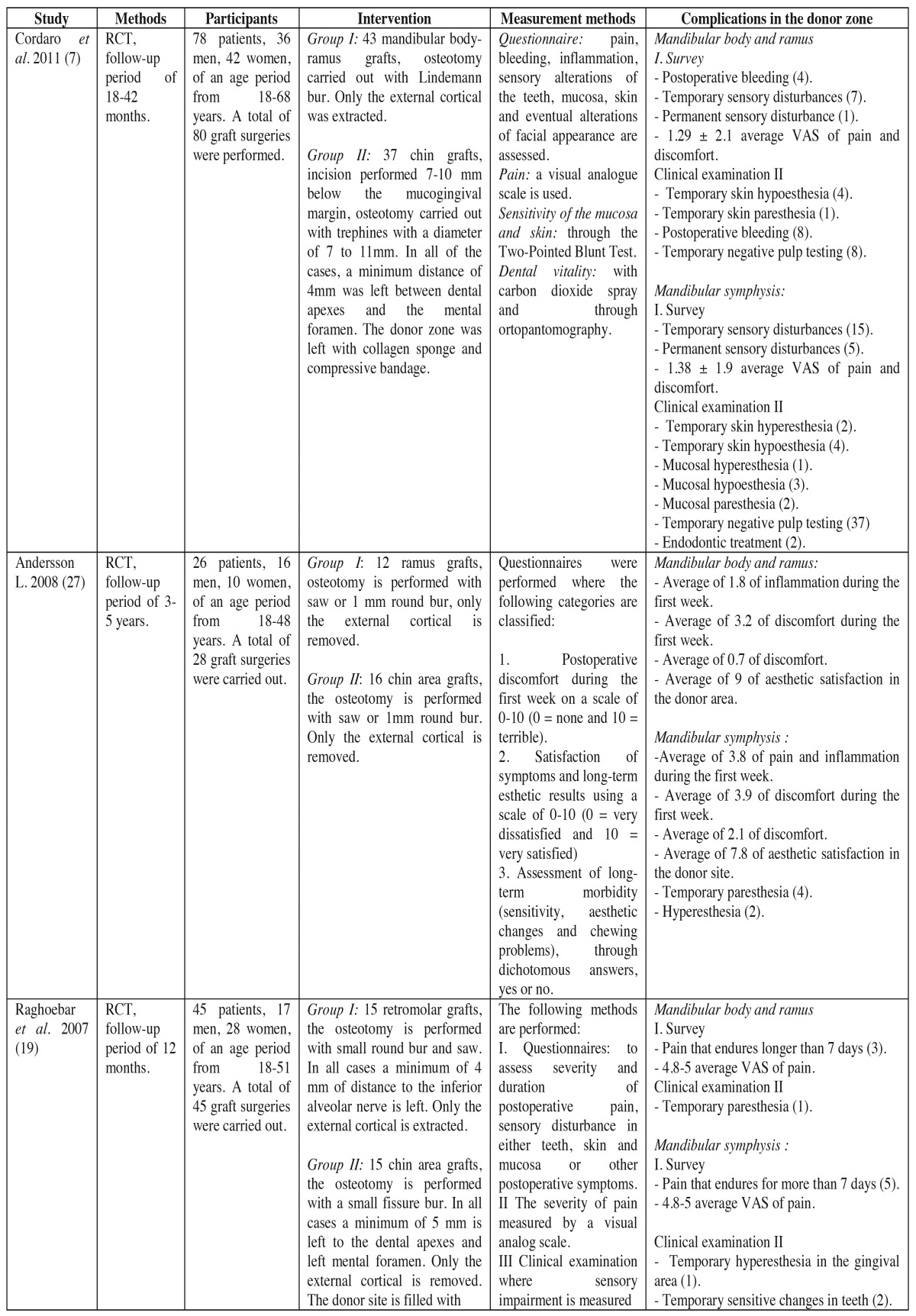
Methods, participants, interventions and results of included studies (RCT = randomized clinical trial, NRCT = non-randomized clinical trial).

**Table 2continue:**
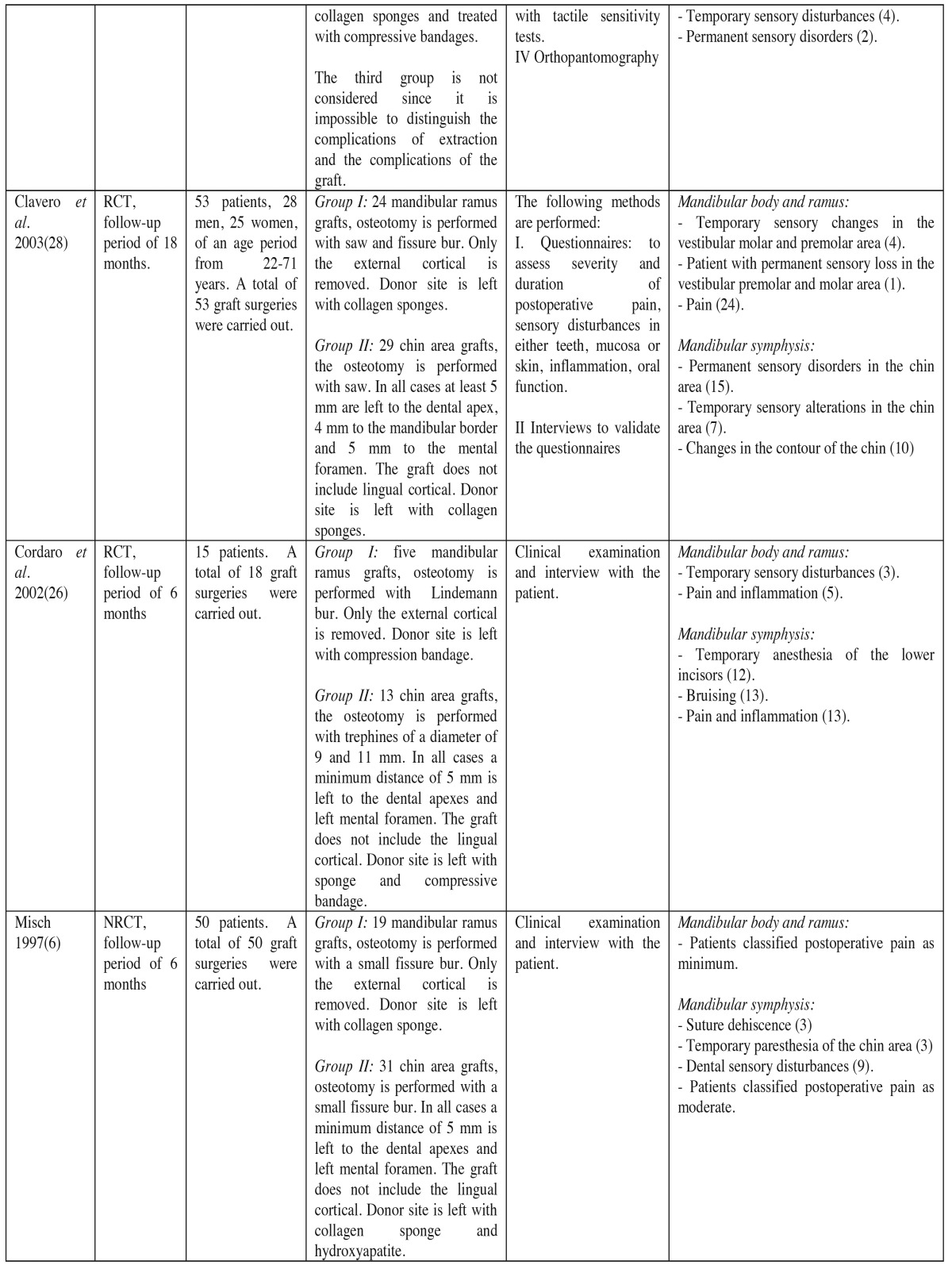
Methods, participants, interventions and results of included studies (RCT = randomized clinical trial, NRCT = non-randomized clinical trial).

A total of 252 patients were treated ( Table 2 and  Table 2continue) (in reality, there were 267, as 15 patients from the study conducted by Raghoebar *et al*. (19) were not considered, given the impossibility of distinguishing complications specific to graft surgery from those related to third molar extractions). Of the 252 intervened patients, 259 underwent bone graft surgeries, 118 of which were mandibular body and ramus grafts, and 141, symphysis grafts. In regards to the ramus grafts, in 67 cases, burs were used to undertake the osteotomy procedure (using Lindeman burs, fissure burs, or round burs); in 51 cases the osteotomy was performed with a saw and round bur or fissure bur generally used for the lower osteotomy procedure. In 118 cases, the osteotomy was performed only in the cortical bone. In 43 cases collagen sponges were left in the site, and in 5 cases compression bandages were used, leaving the remaining patients with no treatment. Regarding the chin area grafts, in 46 cases the graft was extracted with fissure burs, in 50 cases trephines with diameters between 7 and 11 mm were used, and a saw was used in 45 cases.

Of the extracted grafts, 31 corresponded solely to cortical grafts, and in 110, to cortical and medullary tissue.

On 65 occasions the donor site was treated with gelatin sponge and external pressure dressings, on 31 hydroxyapatite and gelatin sponge was used, and in 29 cases only collagen sponges were used; the remaining patients were left untreated.

Regarding the main complications of the mandibular body and ramus donor site; a total of 8.19% of complications (TC) included sensory alterations of the skin and mucosa, followed by postoperative bleeding with 6.55% of TC, 4.37% of TC came as a result of temporary teeth sensory disturbances and permanent sensory disturbances with 1.09% of TC. As for the complications occurred in the symphysis donor site, the most frequent were those associated to temporary sensory disturbances of the teeth reaching 33.87% of TC, followed by the temporary sensory disturbances of the skin and mucosa with 18.57% of TC, permanent sensory disturbances with 12.02% TC, bruising with 7.1% of TC, blemishes with 5.46% of TC, wound dehiscence with 1.63% of TC, and finally, pulp necrosis with 1.09% of TC.  Table 2 summarizes the included articles, describing the design of the study, the patients treated, the analyzed variables, the complications that occurred and the methodology applied when measuring the complications.  Table 3 shows the quality criteria used to analyze the included studies, which can conclude the high risk of bias that the studies show.

**Table 3:**
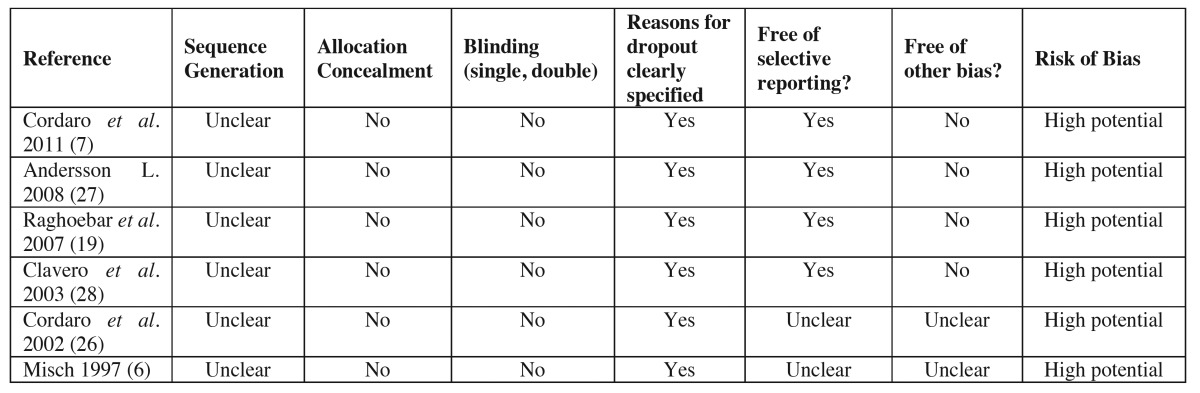
Quality assessment of selected studies.

## Discussion

Despite the different existing alternatives, the autologous bone graft still remains as the chosen graft for bone volume restoration procedures (24) being quality, quantity and high clinical predictability the main reasons that make this type of graft the favorite among the rest of alternatives.

However, one of the major disadvantages of this type of graft is the donor site morbidity. This phenomenon, on the contrary, does not occur with other different types of bone substitutes, being this their main advantage. These bone substitutes have excellent clinical results in small flaws, but in larger defects their predictability is reduced, reason why the autologous bone graft is considered as the first treatment option (25).

The most commonly used intraoral autologous grafts are those that originate from the mandibular body and ramus and symphysis areas, which, according to the analyzed studies, have the following different types and complications that can be divided into 4 groups:

1.- Pain, discomfort and aesthetic results

Although both techniques lack in postoperative pain, classified as slight to moderate, all authors agree that the symphysis graft is more uncomfortable for the patient. The reviewed studies show a maximum of 7 days of pain persistence, which completely disappears in 30 days, in both mandibular body and ramus graft and the symphysis graft. Studies conducted by Cordaro *et al*. (7,26) do not show significant differences when it comes to pain and degree of discomfort when comparing both types of grafts, being in both cases classified as mild. On the contrary, Andersenn (27) found statistically significant results when it comes to more postoperative pain and discomfort when using the mandibular symphysis graft. Regarding discomfort, Clavero and Lundgren (28) detail more difficulties for the patients in the mandibular body and ramus group when speaking, eating and drinking compared to the symphysis group.

Other factors to be considered are the changes in the facial contour that follow the bone graft surgery. Clavero and Lundgren (28) reported that out of the 29 patients that were operated on the symphysis area, 10 had changes in their facial contour. Anderssen (27) also reported aesthetic changes in the symphysis zone. In this study, the operated patients rated their aesthetic satisfaction following the surgery, with an average of 7.8 points (being 10 the highest mark of satisfaction). Other authors such as Nóia *et al*. (29) and Dik *et al*. (30) also described changes in the facial contour in symphysis graft surgery patients. Dik *et al*. (30) noted that the degree of alteration of the facial contour depended on the age of the patient and the size of the obtained graft; older patients with larger bone graft sizes correspond to greater changes in the facial contour. Various authors recommend that to lessen this type of complications, it is advisable to extract the graft from the parasymphysis zone without touching the mandibular symphysis, by not lifting it completely (being the inferior mandibular border the limit), and filling the donor site with bone substitute.

2.- Temporary and permanent sensory alterations of the skin and mucosa

One of the main complications that often cause more discomfort among patients are the sensory disturbances that may occur as a result of obtaining the graft. All the reviewed studies noted a higher prevalence of alterations when using symphysis grafts. Cordaro *et al*. (7) indicate that 40.5% of patients who underwent mandibular symphysis graft surgery showed some type of temporary sensory disturbance, and 13.5% presented a permanent sensory disturbance. By contrast, only 16.2% of the patients treated in the mandibular body and ramus areas had temporary sensory disturbances and 2.3% had permanent sensory disturbances, which shows significant differences between the sites. Andersson (27) described that 25% of the patients that undergo symphysis graft surgery present temporary paresthesia, and none showed alterations when the graft was taken from the mandibular body and ramus areas. Raghoebar *et al*. (19) mention that 46.6% of grafts taken from the symphysis had temporary sensory disturbances and only 6.6% of patients intervened in the body and ramus showed temporary mandibular paresthesia. In either case, no permanent alterations were shown. Clavero *et al*. (28) mention that 51.7% of patients operated on the symphysis area had permanent alterations and 24.13% temporary alterations; however, in the body and ramus areas, 16.6% of patients experienced temporary alterations, leaving 4.1% with permanent alterations. Misch (6) noted that 9.6% of the symphysis-operated patients showed temporary sensory disturbances, without any permanent alterations, and none at the ramus area.

When comparing studies, there is not a single feature that leads to deduce or explain why some studies have found a higher percentage of paresthesia of the symphysis than others. A risk factor to be considered is the type of incision to access the area. Gapski *et al*. (20) point out that using the crevicular incision with two posterior incisions to the mental foramen produces minimal trauma of the mental nerve. Another risk factor to contemplate is the extension of the area to be removed and trauma to the mental nerve that follows the separation of the area.

3. Dental vitality 

Another type of complication that frequently occurs relates to dental sensory alterations, which can be temporary or permanent (corresponding in the latter to pulp necrosis). Tooth sensory alterations using the mandibular body and ramus graft correspond to a total of 8 cases, being all temporary and described only by Cordaro *et al*. (7) This study does not mention the possible causes of these disorders, being the surgical technique similar to that of other articles analyzed.

By using the chin area as the donor site, in 64 cases there were sensory disturbances of the lower anterior teeth; of these, 2 corresponded to pulp necrosis and 62 to temporary sensory disturbances. Cordaro *et al*. (7) are the only ones that describe the states of pulp necrosis. When comparing the surgical procedure performed in this study to those carried out in other studies, the main difference lies in the safety margin left to the dental apex. Cordaro *et al*. (7) consider this safety margin 4 mm, while other studies leave a margin of 5 mm. On temporary sensory disturbances, *Cordaro et al*. (7) presented the most alterations with 37 cases, followed by *Cordaro et al*. (26) with 12 patients, Misch (6) with 9 patients, Raghoebar *et al*. (19) with 2 and Andersson (27) also with 2. The reviewed articles do not mention the exact period during which the normal sensitivity of the affected teeth was recovered.

4. Other complications

The reviewed studies also describe other types of complications, which present a low prevalence. Cordaro *et al*. (7) indicated 12 cases of postoperative bleeding using the mandibular body and ramus graft; on the contrary, no studies report postoperative bleeding when using grafts originated from the chin area. Another complication which occurred only at the symphysis and described only by Cordaro *et al* . (26), which occurred in 13 cases, was the presence of bruising. Finally, Misch (6) describes as a complication the presence of wound dehiscence, which occurred in 3 cases.

In conclusion, it can be said that despite the small number of investigations which comply with the selection criteria included in the review, it appears that the majority of complications occur when using the chin area as the donor site. This area, as well as presenting a higher prevalence and severity of complications, generates greater discomfort for the patient, showing high levels of sensory disturbances in teeth, skin and mucosa, both temporary and permanent, in addition to aesthetic changes, despite being the latter rare.

Thus, it is necessary to carry out a greater number of randomized clinical trials in the future, assessing a larger number of patients, along with the possible complications. It is recommended that clinical trials detail sensory recovery periods and the type of treatment used for recovery.
